# Mixed-microbe solid-state fermentation modulates the physicochemical and aroma profiles of *Eucommia ulmoides* leaf

**DOI:** 10.3389/fmicb.2026.1844724

**Published:** 2026-05-29

**Authors:** Bingnan Gu, Wei Zhang, Bing Guo, Xin Wang, Faliang Wu, Yuheng Zhang, Hailong Tian, Xingke Li, Jihong Huang

**Affiliations:** 1 Institute of Microbial Engineering, School of Life Sciences, Henan University, Kaifeng, China; 2 Engineering Research Center for Applied Microbiology of Henan Province, Kaifeng, China; 3 Henan Key Laboratory of Synthetic Biology and Biomanufacturing, Kaifeng, China; 4 Henan Sanweigi Food Limited Liability Company, Sanmenxia, China; 5 Agricultral College, Henan University, Kaifeng, China

**Keywords:** *Eucommia ulmoides* leaf, functional ingredients, metabolomics, mixed-bacteria solid-state fermentation, volatile flavor

## Abstract

**Introduction:**

*Eucommia ulmoides* leaf (EL) is rich in diverse bioactive constituents.

**Methods:**

This study employed solid-state fermentation with *Lactiplantibacillus plantarum* and *Bacillus subtilis* to improve its quality and flavor. Changes in functional components, metabolites and flavor profiles were analyzed pre-and post-fermentation. Untargeted metabolomics based on UHPLC-MS and HS-SPME-GC-MS revealed 1,698 non-volatile and 827 volatile metabolites in EL and fermented EL.

**Results:**

Analysis of differential metabolites indicated that solid-state fermentation significantly modulated key metabolic pathways, including phenylpropanoid biosynthesis, carbohydrate metabolism, and amino acid metabolism. This metabolic reprogramming enriched the profile of pharmacologically active metabolites such as amino acids, peptides, flavonoids, and alkaloids, and altered the content of specific bioactive compounds: geniposidic acid and chlorogenic acid increased, whereas total flavonoids, polyphenols, and polysaccharides decreased. Flavor analysis revealed that 2-methylisoborneol, the key substance of earthy and musty flavor, was significantly reduced after fermentation, while the contribution of 2-sec-butyl-3-methoxypyrazine and damascenone, the characteristic aroma components of green leaf and fruit flavor, was increased.

**Discussion:**

These shifts collectively resulted in an improved overall flavor profile. This study establishes a theoretical foundation and technical framework for quality and flavor modulation of EL through solid-state fermentation with *Lactiplantibacillus plantarum* and *Bacillus subtilis*, supporting the development of functional EL products.

## 1 Introduction

*Eucommia ulmoides* leaf (EL), a traditional Chinese medicine recorded in the *Chinese Pharmacopeia*, is recognized for its dual potential as both a medicinal and edible herb (Meng et al., 2022). It contains diverse bioactive constituents, including lignans, flavonoids, phenylpropanoids, as well as water-soluble polysaccharides, amino acids, vitamins, and trace elements (Xing et al., 2020). In 2023, EL was listed in the *Catalog of Substances Managed as Both Traditional Food and Medicinal Materials*. Research indicates that EL exhibits antioxidant, antihypertensive, hypolipidemic, hypoglycemic, immunomodulatory, and cardioprotective effects, and has potential to be used as a functional food ingredient (Cai et al., 2024; Ishimitsu et al., 2021; Wu et al., 2023; Zhang et al., 2023). However, the application of EL is constrained by the low bioavailability of certain active constituents and the influence of processing methods on their efficacy (Wu et al., 2023). Furthermore, its inherent bitter and earthy taste presents a significant barrier to consumer acceptance in food product development (Yang et al., 2024).

Solid-state fermentation (SSF) is a green bioprocessing technology that enhances the physicochemical properties and enriches the active components of substrate through microbial enzymatic digestion (Anjos et al., 2025). Current fermentation of EL primarily relies on natural fermentation or single-strain cultivation. In practical production, such fermentation modes are prone to inconsistent product quality and limited process efficiency under conventional operating conditions (Song et al., 2024; Yang et al., 2023). In contrast, mixed-strain fermentation supports refined process regulation and facilitates the biosynthesis of distinctive metabolites that are rarely generated in pure culture systems (Selegato and Castro-Gamboa, 2023). This synergistic effect has also been observed in dark tea co-fermentation, where microbial interactions drive flavor development (Gao et al., 2025). *Lactiplantibacillus plantarum* and *Bacillus subtilis* are widely employed as probiotic fermentation microorganisms. In the fermentation of traditional Chinese medicine, *Lactiplantibacillus plantarum* primarily modulates the synthesis of secondary metabolites, while *Bacillus subtilis* secretes fiber-degrading enzymes, demonstrating synergistic potential in substrate transformation. This synergistic interaction promotes the conversion and release of bioactive constituents, enhances the overall efficacy and ultimately facilitates the generation of beneficial compounds (Yang et al., 2023; Zhang et al., 2023). Previous research indicates that the combination of *Lactiplantibacillus plantarum* and *Bacillus subtilis* in SSF establishes a synergistic interaction that enhances both the conversion and dissolution of bioactive constituents, thereby improving production efficiency (Wang et al., 2025a).

Current studies predominantly focus on the bioactivity of fermented herbs, leaving a gap in the comparative analysis of active ingredients between raw and fermented materials and in the elucidation of their underlying mechanisms, which calls for more in-depth investigation (Luo et al., 2024). Untargeted metabolomics permits the unbiased and comprehensive profiling of all metabolites present in a sample, encompassing unidentified species. This capability renders it a powerful tool for the extensive discovery of novel metabolic pathways, biomarkers, and potential therapeutic targets (Niu et al., 2025). Liquid Chromatography-Mass Spectrometry (LC-MS) forms the analytical foundation of metabolomics. It facilitates the highly sensitive profiling of a broad spectrum of metabolites, including amino acids, amines, and secondary metabolites, thereby constituting a scientific foundation for investigating the mechanistic alterations, functional alterations, and safety implications of fermentation in Chinese herbal medicines (Patti et al., 2012). Wang employed ultra performance liquid chromatography-tandem mass spectrometry coupled with an untargeted metabolomics approach to monitor the dynamic changes in metabolites during saffron petal fermentation (Wang et al., 2024). Headspace solid-phase microextraction coupled with gas chromatography-mass spectrometry (HS-SPME-GC-MS) specializes in capturing the dynamic changes in volatile flavor compounds. The combination of these two techniques enables comprehensive coverage of the metabolite transformation profile throughout the fermentation process. In addition, intelligent sensory technologies such as the electronic nose and the electronic tongue can objectively and quantitatively characterize the overall flavor and taste profiles of samples, serving as direct evidence for assessing improvements in sensory quality (Chen et al., 2024). Metabolomics aims to comprehensively profile the complete set of metabolites within a biological system under specific conditions, which is pivotal for elucidating the active components of fermented drugs and their mechanisms of action (Wang et al., 2006). Zhang employed untargeted metabolomics to monitor differential metabolites in *Platycodon grandiflorus* and *Lycium barbarum* wine across different fermentation durations (Zhang et al., 2024).

In summary, this study employed an integrated multi-omics strategy (LC-MS, HS-SPME-GC-MS, and electronic sensing) to investigate the effects of mixed-culture SSF with *Lactiplantibacillus plantarum* and *Bacillus subtilis* on EL. The fermentation process effectively remodeled the profile of bioactive component and improved the aroma of EL by reducing its bitterness and earthiness. Key differential metabolites and metabolic pathways were identified, thereby laying a mechanistic foundation for the development of high-value functional products from EL.

## 2 Materials and methods

### 2.1 Reagents and chemical standards

EL used in this study was harvested from fresh leaves of *Eucommia ulmoides* trees on the campus of Henan University in Kaifeng City, Henan Province, China, with a harvesting period from September to October. *Lactiplantibacillus plantarum* was provided by the Fermentation Engineering Laboratory of Henan University, while *Bacillus subtilis* was purchased from Beijing Solarbio Science and Technology Co. Ltd. and stored at −80 °C in a laboratory refrigerator. Formic acid, acetonitrile, rutin (RU), gallic acid, geniposidic acid (GPA), chlorogenic acid (CA), and pinoresinol diglucoside (PDG) were purchased from Beijing Solarbio Technology Co., Ltd. Methanol was purchased from Hebei Siyou Excellence Technology Co., Ltd.

### 2.2 Fermentation and sample preparation of EL

#### 2.2.1 Bacterial strain activation

MRS and LB media were prepared as described (Yao et al., 2018). *Lactiplantibacillus plantarum* and *Bacillus subtilis* strains, preserved at −80 °C in glycerol stocks, were revived by inoculating (2% v/v) into MRS and LB liquid media, respectively, followed by incubation at 37 °C with shaking at 200 rpm for 24 h. For bacterial suspension preparation, the seed cultures were centrifuged (9,100 g, 15 min, 4 °C). The resulting pellets were washed twice sequentially with sterile PBS and deionized water. The final suspensions of *Lactiplantibacillus plantarum* and *Bacillus subtilis* were adjusted to approximately 1.17 × 10^8^ and 1.21 × 10^8^ CFU/mL, respectively, for subsequent experiments.

#### 2.2.2 Fermentation of EL

According to the study on SSF of EL using mixed bacterial cultures by Wang with minor modifications (Wang et al., 2025a), 10 g of EL was weighed and placed in a sterilized large Petri dish under aseptic conditions. The *Lactiplantibacillus plantarum* (1.17 × 10^8^ CFU/mL) and *Bacillus subtilis* (1.21 × 10^8^ CFU/mL) strains was mixed at a ratio of 1:3, with a total inoculum volume of 35% (v/w). The fermentation was carried out in a constant-temperature incubator at 25 °C in darkness. The initial moisture content was set at 25% and the total fermentation time was 33 h. After fermentation, the product was dried at 45 °C and ground into a fine powder. Subsequently, 0.1 g each of EL and FEL were accurately weighed separately. For each sample, 2.5 mL of distilled water was added. The mixture was then thoroughly vortexed to ensure complete homogenization and allowed to stand for 30 min. Ultrasonic extraction was then conducted for 30 min, after which the mixture was centrifuged at 8,000 r/min and 4 °C for 10 min. The resulting supernatant was collected, filtered through a 0.22 μm membrane filter, and stored for subsequent analysis.

### 2.3 Determination of chemical composition

#### 2.3.1 Determination of total flavonoid (TF)

According to the method described by Halim et al. (2022), a standard calibration curve was established using rutin as the reference substance. The absorbance was measured at 510 nm, and the linear regression equation was obtained as: y = 0.5475x + 0.0509, *R*^2^ = 0.9929, with a linear range of 0.3–1.0 mg/mL. The sample solution prepared in Section 2.2.2 was diluted 20-fold, and the TF concentration was determined and calculated in accordance with the above method.

#### 2.3.2 Determination of total polyphenol (TPP)

The total polyphenol content of the samples was determined using the Folin-Ciocalteu method (Lohvina et al., 2022). A standard curve was constructed with gallic acid as the reference compound. The absorbance was measured at 765 nm, and the linear regression equation was obtained as follows: y = 0.0146x + 0.0752, *R*^2^ = 0.9997, with a linear range of 10–45 μg/mL. The sample solution prepared in Section 2.2.2 was diluted 80-fold, and the TPP concentration was quantified in accordance with the above method.

#### 2.3.3 Determination of total polysaccharide content (TPS)

The TPS content was determined according to the phenol-sulfuric acid method described by Peng et al. (2022). A standard curve was constructed with glucose as the reference substance, and the absorbance was measured at 490 nm. The linear regression equation was obtained as follows: y = 8.531x + 0.0966, *R*^2^ = 0.9993, with a linear range of 0–0.14 mg/mL. The sample solution prepared in Section 2.2.2 was diluted 160-fold, and the TPS concentration was then determined and calculated based on the above standard curve.

#### 2.3.4 Determination of GPA, CA, RU, and PDG content

The concentrations of GPA, CA, RU and PDG in EL before and after fermentation were quantified using high-performance liquid chromatography (HPLC). Standard solutions of each compound were prepared in methanol at concentrations ranging from 0.05 to 1 g/L. Separation was performed on a Welch Boltimate C18 column (100 mm × 4.6 mm, 2.7 μm) maintained at 25 °C. The mobile phase consisted of acetonitrile (A) and aqueous 0.1% formic acid (B) at a flow rate of 1.0 mL/min. The gradient elution program was set as follows: 0–12 min, 5% to 8% A; 12–20 min, 8% to 15% A; 20–30 min, 15% to 20% A; 30–40 min, 20% to 30% A; 40–45 min, 30% to 5% A; 45–47 min, 5% A (isocratic). Under the established chromatographic conditions, each sample solution was injected at a volume of 10 μL and detected at wavelengths of 238 nm and 194 nm. The corresponding regression equations are presented in Table 1.

**Table 1:** Regression equations of GPA, CA, RU, and PDG.

Bioactive compounds	regression equations	*R* ^2^
GPA	y = 0.1648x + 0.0007	0.9998
CA	y = 0.2501x + 0.0005	0.9997
RU	y = 0.1500x + 0.0014	0.9998
PDG	y = 0.0471x + 0.0026	0.9985

### 2.4 Determination by electronic nose and electronic tongue

#### 2.4.1 Electronic nose detection

3 g of sample was taken and sealed in a 50 mL centrifuge tube for 30 min for headspace sampling detection. The detection conditions were as follows: sampling time was 1 s/group; the self-cleaning time of the sensor was 60 s. The sample injection time was 5 s; the injection flow rate was 400 mL/min. The analysis sampling time was 80 s. The data of 69–71 s were taken as the results for summary and analysis. The PEN3 electronic nose system employed in this study comprises 10 sensors, whose corresponding sensitive substances are listed in Table 2.

**Table 2:** PEN3 electronic nose sensor sensitive substances.

Sensor number	Sensor code	Sensitive substances
1	W1C	Aromatic hydrocarbons
2	W5S	Nitrogen oxides
3	W3C	Ammonia; aromatic molecules
4	W6S	Hydrides
5	W5C	Olefins, aromatics and polar molecules
6	W1S	Alkanes
7	W1W	Sulfur compounds
8	W2S	Alcohols and partial aromatic compounds
9	W2W	Aromatic hydrocarbons and organosulfur compounds
10	W3S	Alkanes and aliphatic compounds

#### 2.4.2 Electronic tongue detection

The sensor was cleaned with cleaning solution and the reference solution to the equilibrium position for 30 s, and the sample solution was diluted to 0.04 g/mL for on-machine testing. The measurement lasted for 30 s, and the initial taste values were recorded. Subsequently, the sensor was cleaned with reference solution for 3 s and then transferred to a fresh reference solution to measure the aftertaste for an additional 30 s. The five food taste sensors C00 (acidic bitterness), AE1 (astringency), CA0 (sourness), CT0 (salty), AAE (umami) were used for four repeated measurement cycles. The first cycle was discarded, and the average of the last three measurements was taken as the final result.

### 2.5 Untargeted metabolomics analysis

#### 2.5.1 LC-MS profiling of non-volatile metabolites

50 mg of EL sample was weighed, and 400 μL of extraction solvent (methanol: water = 4:1) was added. The mixture was ground for 6 min using a frozen tissue grinder, followed by low-temperature ultrasonic extraction at 5 °C and 40 kHz for 30 min, and then statically placed at −20 °C for 30 min. After centrifugation at 4 °C and 13,000 r/min for 15 min, the supernatant was transferred to a sample vial for subsequent analysis. Liquid phase conditions: Chromatographic column: Waters ACQUITY UPLC HSS T3 (100 mm × 2.1 mm i.d., 1.8 μm); Injection volume: 3 μL; Column temperature: 40 °C; Mobile phase: A 95% water + 5% acetonitrile (containing 0.1% formic acid)—B 47.5% acetonitrile + 47.5% isopropanol + 5% water (containing 0.1% formic acid); Gradient elution program (0–3 min, 100% A−0%B; 3–9 min, 80%A−20%B; 9–11 min, 40%A−60%B; 11–13.6 min, 0%A−100%B; 13.6–16 min, 100% A−0%B). Mass spectrometry conditions: the mass spectrometry signal was in positive and negative ion scanning mode [ESI (+), ESI (–)]; spray voltage 3.5 kV; heating temperature 425 °C; capillary temperature 325 °C; mass spectrometry scanning range 70–1,050 m/z; resolution 60,000.

#### 2.5.2 HS-SPME/GC-MS profiling of volatile metabolites

Accurately weighed 3.0 g of EL sample was placed in a 20 mL headspace vial, sealed and subsequently analyzed using a headspace solid-phase microextraction (HS-SPME) coupled with gas chromatography/mass spectrometry (GC/MS) system. The headspace conditions were as follows: 130 °C in the heating chamber, 150 °C in the quantification loop, 170 °C in the transfer line, 20 min of sample equilibrium, 0.5 min of injection, 35 min of cycling, and 10 psi (helium-driven) in the quantification loop. Gas chromatographic analysis was performed on a VF-WAXms capillary column (Agilent CP9204, 25 m × 0.25 mm × 0.2 μm) with an inlet temperature of 180 °C, high-purity helium carrier gas of 2 mL/min, a spacer pad purging of 3 mL/min, and a split-injection mode (split ratio of 10:1, injection volume of 1 μL). The initial oven temperature was held at 40 °C for 2 min, ramped to 100 °C at 5 °C/min, then ramped to 230 °C at 15 °C/min and maintained for 5 min, followed by a post-run hold at 230 °C for 2 min. Mass spectrometry was performed using an electron bombardment ion source (EI), with the temperature of the transmission line at 310 °C, the temperature of the ion source at 230 °C, and the temperature of the quadrupole at 150 °C, and the ionization energy at 70 eV and full scan mode (SCAN) covering m/z 30–1,000 at a scan rate of 3.2 scans/s.

### 2.6 Data processing and analysis

All experimental data were collected from three or more independent replicates and are presented as mean ± standard deviation (SD). Statistical analyses were performed using IBM SPSS Statistics (version 27.0.1), and graphs were plotted with Origin 2022. The mass spectrometry data were processed by Progenesis QI software, and metabolite identification was performed by searching public databases (LIPID MAPS, HMDB, Metlin) and Majorbio's own database. The raw data of GC/MS were analyzed by Mass Hunter Workstation Quantitative Analysis software (v10.0.707.0). The metabolites were also searched for library identification using Fiehn and NIST public databases, and the searched data were uploaded to the Meggie BioCloud platform (https://cloud.majorbio.com) for data analysis. Annotation of differential metabolites and metabolic pathway enrichment analysis were conducted using the KEGG database and Majorbio Cloud Tools.

## 3 Results and discussion

### 3.1 Effect of fermentation on the content of main active components of EL

**Figure 1:**
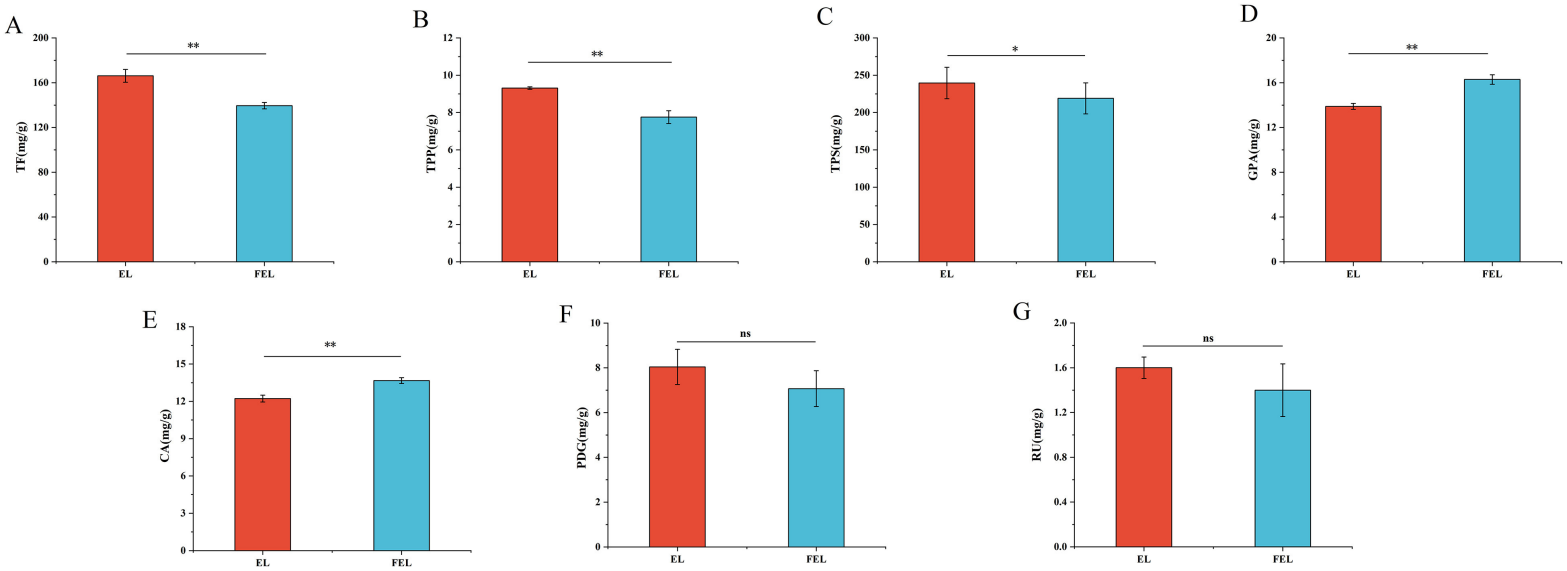
Changes in active components and hypoglycaemic activity of EL and FEL. **(A)** TF content. **(B)** TPP content. **(C)** TPS content. **(D)** GPA content. **(E)** CA content. **(F)** PDG content. **(G)** RU content. *Indicates significant difference, *p* < 0.05; **indicates highly significant difference, *p* < 0.01.

To investigate the dynamic changes in the major bioactive components before and after fermentation, quantitative analyses were conducted in this study for TF, TPP, TPS, GPA, CA, PDG, and RU. As shown in Figure 1, following SSF with *Lactiplantibacillus plantarum* and *Bacillus subtilis*, the contents of TF, TPP, and TPS decreased significantly from 166.15 ± 5.45 mg/g, 9.31 ± 0.19 mg/g, and 240.73 ± 7.50 mg/g to 139.51 ± 2.87 mg/g, 7.76 ± 0.30 mg/g, and 208.69 ± 6.50 mg/g, corresponding to reduction rates of 16.03%, 16.67%, and 16.63%. In contrast, the contents of GPA and CA increased significantly from 13.88 ± 0.29 mg/g and 12.22 ± 0.32 mg/g to 16.28 ± 0.22 mg/g and 13.67 ± 0.10 mg/g, with respective increases of 17.29% and 11.87%. Meanwhile, the contents of PDG and RU decreased from 8.04 ± 0.91 mg/g and 1.60 ± 0.10 mg/g to 7.07 ± 0.85 mg/g and 1.40 ± 0.27 mg/g, respectively, though these changes were not statistically significant. Following fermentation, the levels of TF, TPP and TPS all decreased significantly. This reduction can likely be attributed to the extracellular enzymes (e.g., β-glucosidase, esterase) secreted by *Lactiplantibacillus plantarum* and *Bacillus subtilis* during the process. These enzymes may degrade flavonoids-type catechol structures in EL (Guo et al., 2017), directly break down plant cell-wall polysaccharides such as cellulose and hemicellulose, and further hydrolyze polysaccharide chains through β-glucosidase (Vastano et al., 2016). Additionally, bound polyphenols and flavonoid glycosides may be converted into small molecules or further degraded into metabolites such as quinones and organic acids, leading to an overall reduction in their total content (Filannino et al., 2018). Correspondingly, Yang also reported that mixed fermentation of *Lycium barbarum* leaf with *Lactiplantibacillus plantarum* and *Bacillus subtilis* decreased flavonoid content (Yang et al., 2018). Furthermore, Liu similarly demonstrated that fermentation of black-fruited wild cherry berries by *Lactiplantibacillus plantarum* reduced total phenols, flavonoids and sugars (Liu et al., 2025). In summary, SSF significantly reduced the contents of TF, TPP, and TPS in EL, while markedly increasing the contents of GPA and CA. Although this fermentation strategy also exerted a certain degree of reducing effect on the contents of PDG and RU, the changes in the latter two components were not statistically significant.

### 3.2 Electronic nose and electronic tongue analyses of EL fermentation

#### 3.2.1 Sensory characteristics of electronic nose

**Figure 2:**
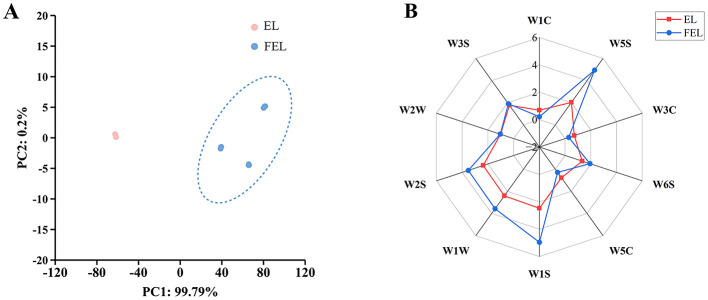
**(A)** PCA and **(B)** Response radar chart of electronic nose for EL before and after fermentation.

Electronic nose analysis showed that there were significant differences in the aroma profile of volatile substances in EL before and after mixed fermentation. Principal component analysis (PCA) revealed that the cumulative contribution rate of PC1 and PC2 reached 99.99% (Figure 2A), which was sufficient to fully represent the overall aroma profile of the samples. Notably, the samples before and after fermentation were distinctly separated into different regions in the PCA score plot, confirming that the electronic nose could effectively distinguish between the two groups. As illustrated in Figure 2B, the responses of W5S (nitrogen oxides), W1S (alkanes), W1W (sulfur compounds), and W2S (aromatic hydrocarbons and organic sulfur compounds) exhibited significant differences between pre-and post-fermentation samples. These findings demonstrated that nitrogen oxides, alkanes, and sulfur compounds were the major differential volatile components in EL subjected to multi-bacteria fermentation. This result is consistent with the conclusion of previous studies that fermentation with different lactic acid bacteria can significantly alter the contents of sulfur-containing compounds, alcohols, aldehydes, ketones, and aromatic flavor components in EL. Combined with the data presented in Table 3, the response values of sensors W1C, W5S, W3C, W6S, W5C, W1S, W1W, W2S, and W3S all exhibited significant changes for the FEL, indicating that multi-strain fermentation had a remarkable impact on the composition of their flavor compounds. Specifically, the contents of polar substances such as aromatic hydrocarbons, ammonia and aromatic molecules, as well as alkenes and aromatics decreased significantly, while those of nitrogen oxides, hydrides, alkanes, sulfur compounds and aliphatic substances increased markedly. The specific composition of these compounds can be further analyzed in depth by combining with volatile metabolomics.

**Table 3:** Response of electronic nose sensors in EL before and after fermentation.

Sensors	EL	FEL
W1C	0.69 ± 0.02^a^	0.21 ± 0.01^b^
W5S	2.03 ± 0.08^a^	4.94 ± 0.29^b^
W3C	0.71 ± 0.02^a^	0.28 ± 0.01^b^
W6S	1.33 ± 0.02^a^	1.93 ± 0.02^b^
W5C	0.77 ± 0.01^a^	0.29 ± 0.01^b^
W1S	2.47 ± 0.11^a^	4.96 ± 0.21^b^
W1W	2.41 ± 0.12^a^	4.57 ± 0.13^b^
W2S	2.37 ± 0.10^a^	3.53 ± 0.17^b^
W2W	1.02 ± 0.01^a^	1.04 ± 0.01^a^
W3S	1.77 ± 0.03^a^	1.90 ± 0.03^b^

Different superscript letters indicate significant differences between groups, p < 0.05.

#### 3.2.2 Sensory characteristics of electronic tongue

**Figure 3:**
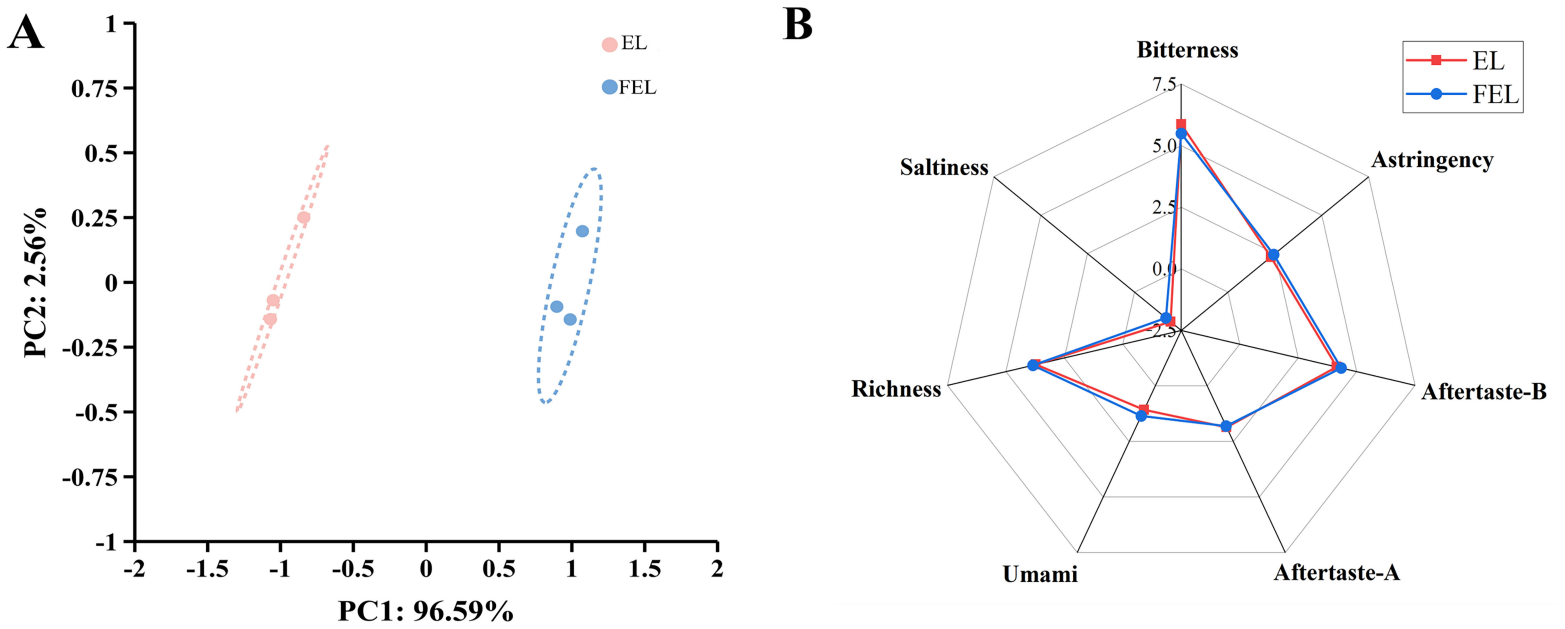
**(A)** PCA of electronic tongue and **(B)** Response radar chart of electronic nose for EL before and after fermentation.

An electronic tongue was employed to simulate the taste buds of the human tongue for the analysis of EL before and after multi-strain fermentation. As shown in Figure 3A, the cumulative contribution rate of the first two principal components (PC1 and PC2) derived from the electronic tongue analysis reached 99.15%. This value exceeded 99%, indicating that these two principal components could sufficiently reflect the main information characteristics of the volatile substances in the multi-strain fermented EL. Combined with the data presented in Figure 3B and Table 4, the sourness values of EL both before and after fermentation were lower than the threshold of tastelessness, indicating the absence of sour taste in the samples regardless of fermentation treatment. Compared with those before fermentation, the bitterness of EL after fermentation decreased significantly by 6.31%, while the umami taste, taste richness, saltiness, and after-bitterness increased significantly by 26.17%, 3.49%, 6.14%, and 4.58%, respectively. The results demonstrated that multi-strain fermentation with *Lactiplantibacillus plantarum* and *Bacillus subtilis* could reduce the bitterness of EL, enhance their umami taste, and thus exert a certain improvement effect on the taste profile of EL.

**Table 4:** Response of electronic tongue sensors in EL before and after fermentation.

Taste	Taste threshold	EL	FEL
Sourness	−13	−28.69 ± 0.07^a^	−30.57 ± 0.04^b^
Bitterness	0	5.86 ± 0.10^a^	5.49 ± 0.08^b^
Astringency	0	2.28 ± 0.06^a^	2.43 ± 0.05^a^
Aftertaste-B	0	4.15 ± 0.06^a^	4.34 ± 0.04^b^
Aftertaste-A	0	1.86 ± 0.03^a^	1.81 ± 0.04^a^
Umami	0	1.07 ± 0.05^a^	1.35 ± 0.05^b^
Richness	0	3.72 ± 0.02^a^	3.85 ± 0.04^a^
Saltiness	−6	−1.93 ± 0.01^a^	−1.68 ± 0.06^b^

Different superscript letters indicate significant differences between groups, p < 0.05.

### 3.3 Identification of non-volatile metabolites in EL and FEL

**Figure 4:**
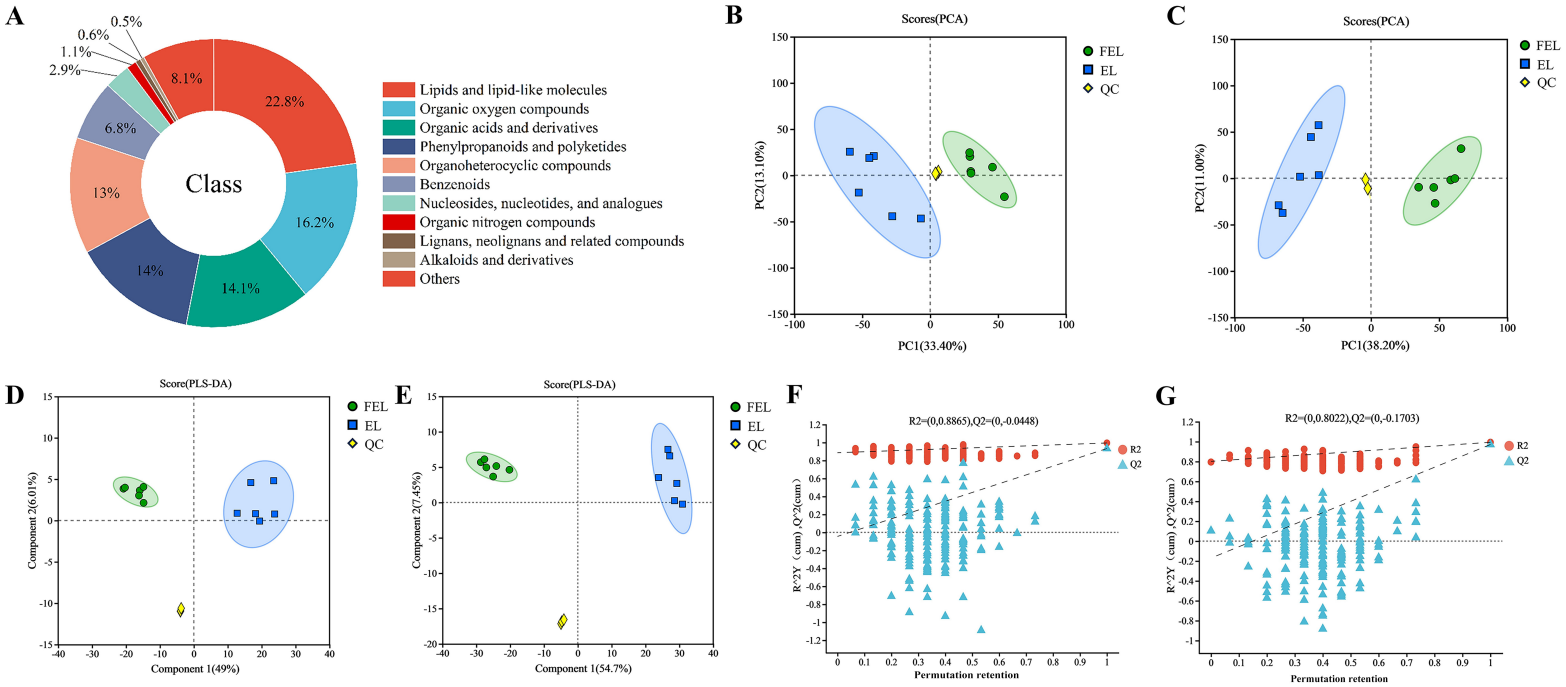
Metabolite identification results before and after FEL. **(A)** Metabolite classification diagram. **(B)** PCA in the negative ion detection mode. **(C)** PCA in the positive ion detection mode. **(D)** PLS-DA score plot in the negative ion detection mode. **(E)** PLS-DA score plot in the positive ion detection mode. **(F)** PLS-DA displacement model in the negative ion detection mode. **(G)** PLS-DA displacement model in the positive ion detection mode.

To investigate the variations in non-volatile metabolites between EL and FEL, non-targeted metabolomics was employed in this study. Twelve samples each of unfermented and fermented leaves were analyzed using both positive and negative ion modes of LC-MS, leading to the identification of 1,698 metabolites. As depicted in Figure 4A, these encompassed 387 Lipids and lipid-like molecules, 275 Organic oxygen compounds, 239 Organic acids and derivatives, 238 Phenylpropanoids and polyketides, 221 Organoheterocyclic compounds, 115 Benzenoids, 49 Nucleosides, nucleotides, and analogs, 19 Organic nitrogen compounds, 10 Lignans, neolignans and related compounds, 8 Alkaloids and derivatives, and 137 other compounds.

PCA results demonstrated (Figure 4B) that the quality control samples exhibited good clustering, indicating reliable bioanalytical performance and data quality. In both positive and negative ion modes, the two sample groups are separable through PCA, with parallel samples clustering together, indicating similar metabolite profiles. The EL and FEL groups exhibited substantial differences. Furthermore, all samples in both positive and negative ion modes fell within the 95% confidence circle in LC-MS analysis, indicating that the data were suitable for subsequent investigation (Cala et al., 2018). The PLS-DA score plot (Figure 4C) further demonstrated distinct separation between the EL and FEL groups. The PLS-DA replacement model (Figure 4D) revealed that both *R*^2^ and *Q*^2^ values gradually decreased with reduced replacement retention, indicating a robust and reliable model without overfitting. Similarly, the PLS-DA score plot in positive ion mode (Figure 4E) also showed clear group separation. Permutation tests further confirmed model validity in both negative (Figure 4F) and positive (Figure 4G) ion modes, with no overfitting observed. In summary,mixed-bacteria SSF significantly altered the non-volatile metabolite composition of EL.

### 3.4 Differential metabolite analysis of EL and FEL

**Figure 5:**
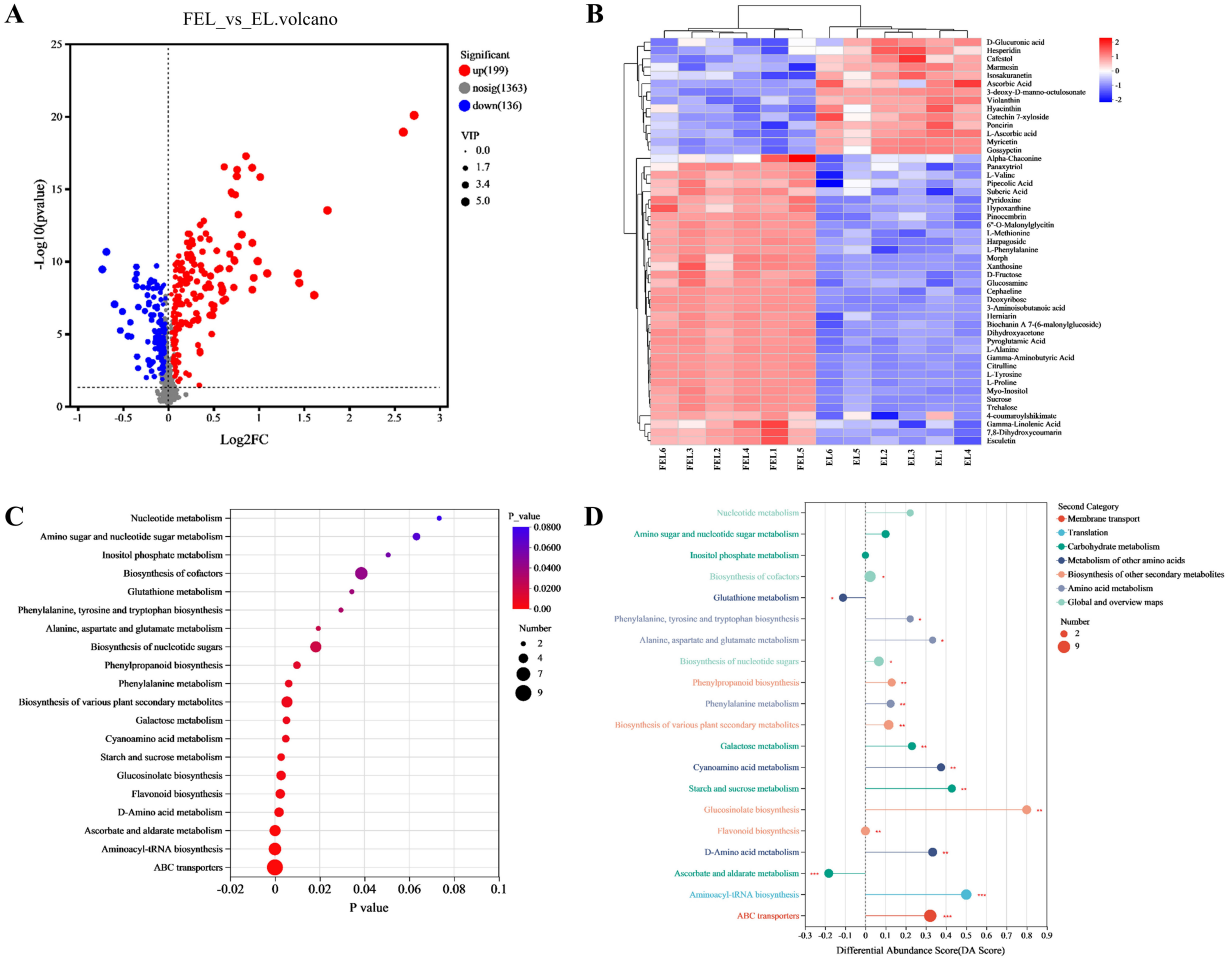
Analysis of differential metabolites in EL and FEL. **(A)** Volcano plot of differential metabolites. **(B)** Clustering heatmap of differential metabolites. **(C)** KEGG pathway enrichment bubble plot for differential metabolites. **(D)** Differential abundance score plot for KEGG pathways in differential metabolites.

The VIP value quantifies the weight of variables within the OPLS-DA model, indicating their influence and explanatory power for distinguishing between treatment groups (Huang et al., 2022). By selecting metabolites satisfying both criteria (VIP > 1 and *p* < 0.05), non-volatile differential metabolites in EL and FEL were identified, and a volcano plot was generated (Figure 5A). A total of 335 differentially expressed metabolites were identified, comprising 199 significantly upregulated metabolites and 136 significantly downregulated metabolites, as detailed in Supplementary Table S1. Key differentially expressed metabolites associated with pharmacological activity were summarized and subjected to cluster analysis (Figure 5B). Post-fermentation analysis revealed significant upregulation of 6 alkaloids, 2 fatty acids, 3 flavonoids, 4 phenylpropanoids, 1 cycloartenolide, 1 organic acid, 1 vitamin, 7 carbohydrates, and 10 peptides. Conversely, 8 flavonoids, 2 carbohydrates, 2 vitamins, 1 terpenoid, and 1 phenylpropanoid were significantly downregulated. These alterations collectively highlight the fermentation process's pivotal role in nutrient degradation and the transformation of bioactive constituents.

#### 3.4.1 Amino acids and peptides

A marked upregulation of various amino acids and their metabolites, including L-alanine, L-tyrosine, L-valine, L-methionine, L-proline, L-phenylalanine, and piperidonic acid, indicates high activity of proteases and peptidases secreted by the fermentative strains, and the fermentation process substantially promotes the degradation of proteins and peptides in EL. Notably, the accumulation of gamma-aminobutyric acid (GABA) suggests an active glutamate decarboxylation pathway within the fermentation system. As a essential neurotransmitter, GABA exhibits anti-stress and sedative effects in animals, and its enrichment substantially enhances the functional value of the fermented products. The results demonstrate that fermentation effectively degrades macromolecular proteins in EL into readily absorbable free amino acids and small-molecule bioactive compounds. This improvement not only improves protein bioavailability but also enhances the physiological regulatory functions of EL.

#### 3.4.2 Carbohydrates

Regarding carbohydrate metabolism, the fermentation process demonstrated thorough utilization and conversion of EL. The upregulation of monosaccharides and oligosaccharides such as D-fructose, glucosamine, trehalose, and sucrose likely resulted from the degradation of complex polysaccharides like pectin and hemicellulose within the leaf cell walls (Liu et al., 2024). This degradation pattern is consistent with the complementary enzymatic properties of the two strains. *Bacillus subtilis* exhibits prominent secretion of cellulase and hemicellulase, which effectively degrade the polysaccharide network of plant cell walls (Ma et al., 2022). Both Baci*llus subtilis* and *Lactiplantibacillus plantarum* possess β-glucosidase activity, which further hydrolyzes oligosaccharides into fermentable monosaccharides (Li et al., 2025; Yu et al., 2025). This sequential “depolymerization followed by hydrolysis” likely accounts for the synchronized upregulation observed across multiple carbohydrate classes. Concurrently, the upregulation of glycolysis intermediate dihydroxyacetone and tricarboxylic acid cycle intermediate succinate reflects vigorous microbial carbohydrate metabolism. This process not only supplies energy for microbial growth but also depletes common anti-nutritional factors in EL, thereby improving their digestibility. Moreover, the upregulation of inositol serves as a positive signal (Ishmayana et al., 2014). This substance possesses vitamin-like effects and prebiotic properties, and its increased content contributes to promoting gut health.

#### 3.4.3 Flavonoids, alkaloids and corresponding derivatives

The increased levels of flavonoids such as phellandrin, 6′-O-succinylgenistein and kaempferol-7-succinylglucoside indicate that the fermentation process significantly influences and enhances the bioactive compound profile of EL. Among these, malonyl-modified flavonoid glycosides typically exhibit greater stability and biological activity; their enrichment likely stems from the biotransformation of glycosides in EL by *Lactiplantibacillus plantarum* and *Bacillus subtilis* (Cho et al., 2011). Concurrently, the upregulation of coumarin compounds such as mappinbarberin and 7,8-dihydroxycoumarin, alongside the polyunsaturated fatty acid γ-linolenic acid, collectively enhanced the antioxidant and anti-inflammatory potential of EL. Conversely, the downregulation of certain flavonoid glycosides, such as catechin-7-xyloside and hesperidin, may result from their hydrolysis into corresponding aglycones by microbial glycosidases. This enzymatic deglycosylation is mediated by β-glycosidase produced by both *Lactiplantibacillus plantarum* and *Bacillus subtilis*, and is of functional significance because aglycones typically exhibit enhanced lipophilicity and potentially improved intestinal absorption compared to their glycosylated precursors (Godse et al., 2025; Kuo et al., 2012; Lee et al., 2022). This conversion is consistent with the previously measured decrease in total flavonoid content.

#### 3.4.4 Organic acids, vitamins, and corresponding derivatives

The downregulation of ascorbic acid (vitamin C) may stem from its instability in the fermentation environment, whereas the upregulation of pyridoxine (vitamin B6) reflects the synthetic contribution of *Lactiplantibacillus plantarum* and *Bacillus subtilis*, enriching the B-vitamin profile. The downregulation of certain secondary metabolites, such as cafestol, may help mitigate undesirable flavors in EL, thereby enhancing their palatability in food processing.

### 3.5 Analysis of metabolic pathways differing between EL and FEL

Differentially expressed metabolites are closely correlated with functional alterations. Analysis of the KEGG pathways enriched by these metabolites primarily map reveals how changes in metabolite levels drive functional shifts, thereby linking these changes to experimental treatments. As depicted in Figure 5C, fermentation exerted a highly significant effect (*p* < 0.001) on differential metabolites involve in ABC transporters, aminoacyl-tRNA synthesis, and ascorbic acid and aldonic acid metabolism pathways. Differential abundance scores were calculated for the metabolic pathways of these differential metabolites, yielding the differential abundance score plot. As depicted in Figure 5D, the vertical axis represents metabolic pathways, while the horizontal axis denotes differential abundance scores. A score of +1 indicates that all annotated differentially expressed metabolites within the pathway exhibit an upregulation trend, whereas −1 signifies a downregulation trend. The size of each dot corresponds to the number of annotated differentially expressed metabolites within the pathway, with larger dots indicating greater metabolic diversity. Among the metabolic pathways associated with differential metabolites, 14 pathways exhibited upregulation. Notably, significant upregulation was observed in thioglucoside biosynthesis, aminoacyl-tRNA synthesis, and starch and sucrose metabolism. Conversely, downregulation was noted in glutathione metabolism and ascorbic acid and aldehyde metabolism.

**Figure 6:**
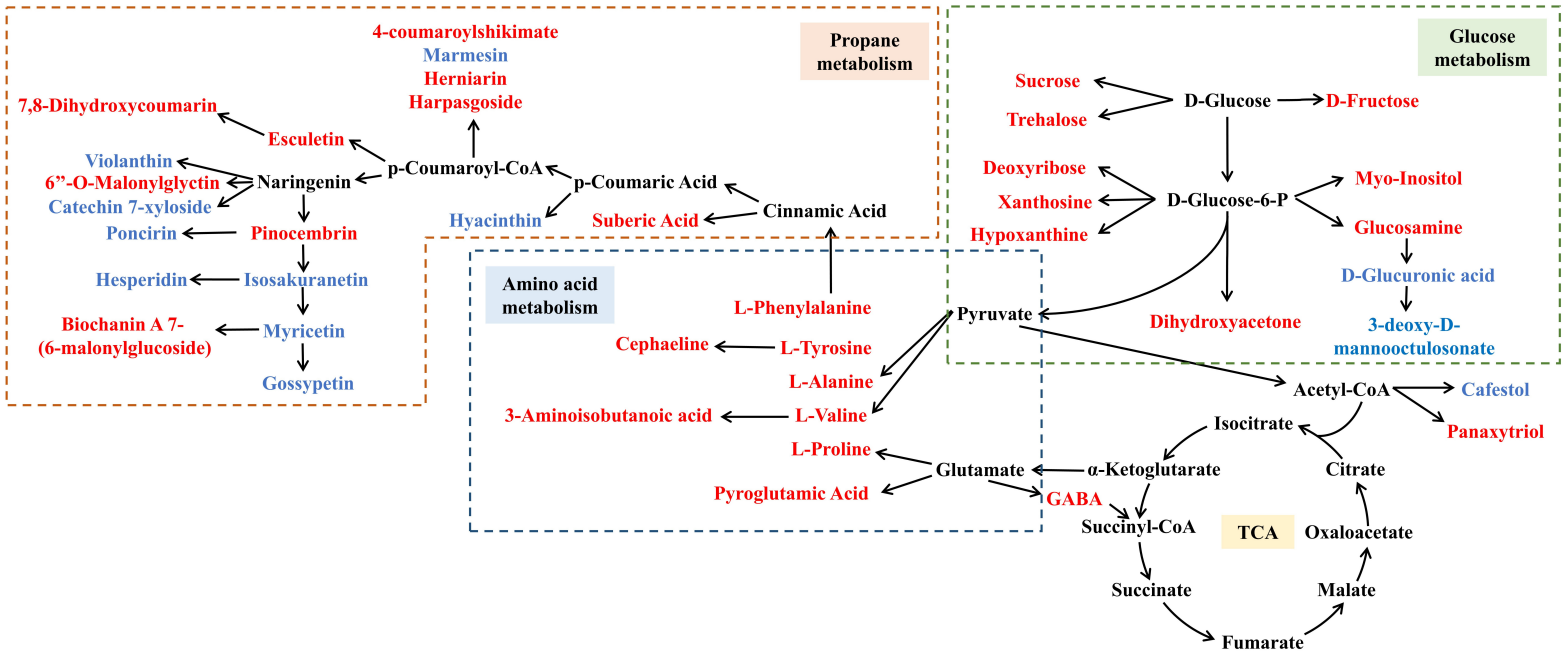
Integrated metabolic pathway diagram and differential metabolite profile of partial metabolism in EL during SSF with *Lactiplantibacillus plantarum* and *Bacillus subtilis* co-cultures. Red indicates upregulation of metabolites, blue indicates downregulation.

The integration of certain metabolic pathways and associated metabolites during fermentation is illustrated in Figure 6. Upregulated metabolites are indicated in red, while downregulated ones appear in blue. Results demonstrate that the phenylpropanoid biosynthesis pathway in EL serves as a pivotal bridge linking primary and secondary metabolism, thereby determining the diversity of plant phenolic compounds. Results indicate that the conversion from L-phenylalanine to p-coumaroyl-CoA constitutes a core step. As a pivotal branching point, regulation of metabolic flux toward coumarin-CoA is crucial. Within EL, this intermediate simultaneously feeds into the synthesis of both coumarin derivatives (e.g., saponin, maemisin) and flavonoids (e.g., naringenin). This multi-branched, synergistic synthesis pattern likely underpins the structural basis for the leaf's rich diversity of phenolic bioactive compounds. Evodia leaves harbor structurally diverse flavonoids, including flavonones like naringenin, flavonols such as myricetin and hesperidin, and malonylglycosides. This structural diversity primarily stems from extensive glycosylation, malonylation, and other modifying reactions. Moreover, the formation of malonylglycosides (e.g., biochanin A 7-malonylglucoside) not only enhances water solubility but is also implicated in plant stress resistance and human bioavailability. Research further indicates that the efficient synthesis of distinctive secondary metabolites in EL heavily relies on carbon skeletons, nitrogen sources, and energy supplied by primary metabolism. The TCA cycle not only serves as an energy source, but its intermediates (such as α-ketoglutarate and oxaloacetic acid) are also prerequisites for amino acid synthesis. Of particular note is the identification of the GABA shunt (γ-aminobutyric acid shunt), which directly links the TCA cycle to amino acid metabolism. As a signaling molecule involved in plant stress responses, GABA accumulation may indirectly regulate phenylpropanoid metabolic activity. This synergistic relationship is also reflected in metabolomic studies of brown rice vs. white rice, where differences in amino acid metabolism significantly influence the composition of downstream flavor compounds and polyphenols. The medicinal value of EL stems not from a single component but from the synergistic action of multiple bioactive constituents. The metabolic networks revealed in this study indicate that phenylpropanoids (flavonoids, coumarins), terpenoids (e.g., cafestol), and alkaloids do not operate in isolation. For instance, flavonoids and ascorbic acid collectively constitute a redox balance system. Terpenoids (e.g., cafestol) and cycloartenol glycosides (e.g., harpagoside) may contribute to the overall physiological activity of EL through alternative mechanisms.

### 3.6 Analysis of volatile metabolites in EL and FEL

**Figure 7:**
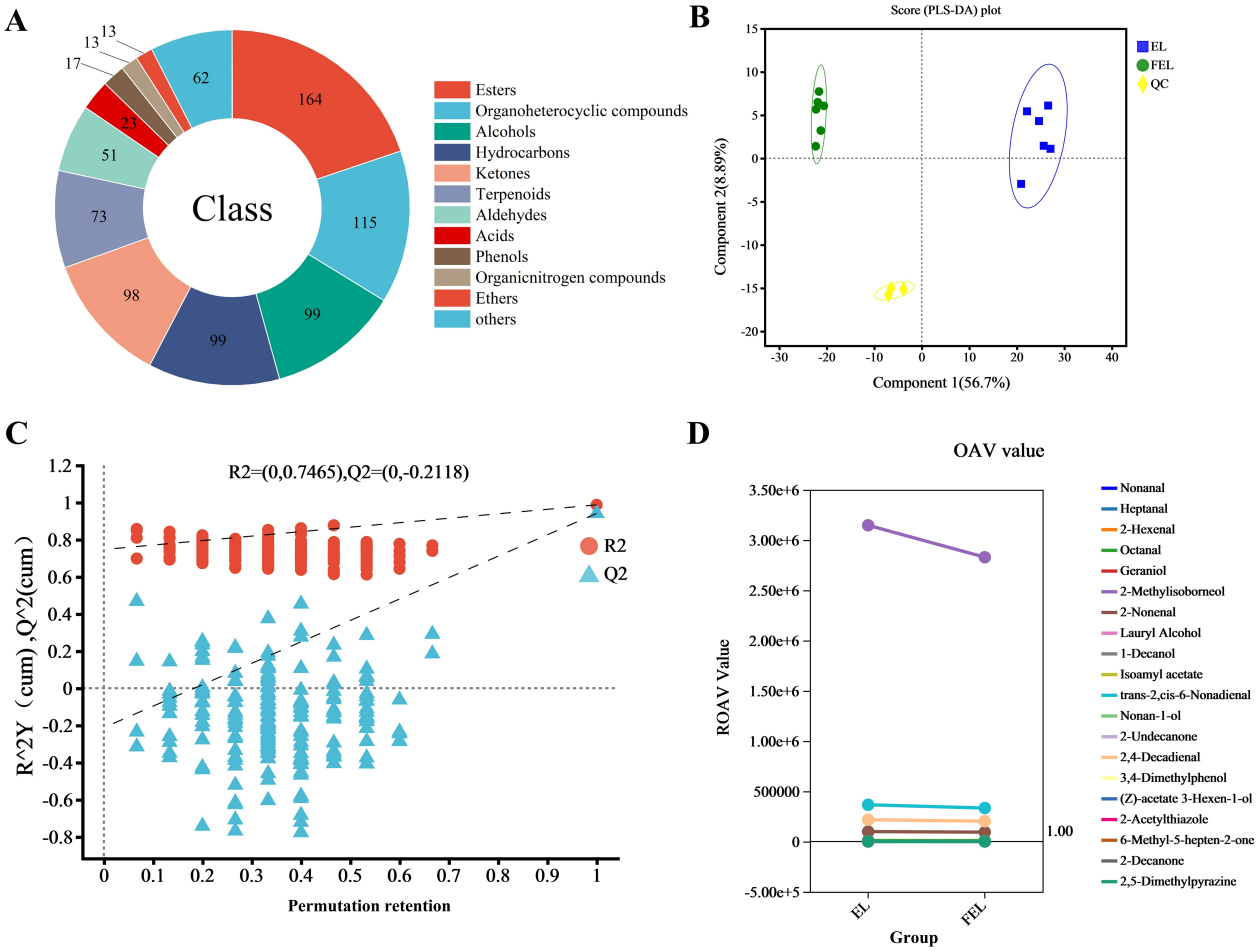
Analysis of volatile metabolites in EL and FEL. **(A)** Classification statistics of volatile metabolites. **(B)** PLS-DA score plot. **(C)** PLS-DA displacement model. **(D)** Odor activity analysis of differential metabolites.

Using HS-SPME-GC-MS, volatile compounds from the SSF of EL by a mixed culture of *Lactiplantibacillus plantarum* and *Bacillus subtilis* were analyzed. A total of 827 volatile flavor compounds were identified, as shown in Figure 7A, including 164 esters, 115 organic heterocyclic compounds, 99 alcohols, 99 hydrocarbons, 98 ketones, 73 terpenes, 51 aldehydes, 23 acids, 17 phenols, 13 nitrogen-containing organic compounds, 13 ethers, and 62 other classes. The PLS-DA score plot illustrates the model's classification efficacy, where greater dispersion of sample points indicates more pronounced separation. As depicted in Figure 7B, the distribution of sample points demonstrates significant classification between pre-fermentation and post-fermentation samples. Figure 7C shows that both *R*^2^ and *Q*^2^ gradually decrease with reduced displacement retention, indicating a robust and reliable model free from overfitting.

The contribution of individual volatile compounds to the overall aroma of EL depends on their concentration and odor threshold, while their contribution is determined by their Odor Activity Value (OAV). Compounds with an OAV > 1 are typically considered key aroma components, with higher OAV values indicating greater contribution to the aroma profile (Jia et al., 2025). Table 5 presents the principal characteristic volatile compounds of EL before and after fermentation, along with the aroma contribution index (ACI) for each compound. ACI is defined as the percentage of a compound's aroma profile threshold relative to the sum of the thresholds of all compounds. Although the classification of volatile compounds in EL remained stable before and after fermentation, its principal characteristic aromas underwent significant alterations. Specifically, the ACI of 2-sec-butyl-3-methoxypyrazine (grassy note) in unfermented EL was 35.08%, while dammarone (apple note) was 30.89% rising to 37.73% and 31.16% post-fermentation respectively. These compounds constituted the primary aroma sources in EL before and after fermentation, indicating their crucial role in imparting characteristic aroma profiles. Neta identified 2-sec-butyl-3-methoxypyrazine and 2-isopropyl-3-methoxypyrazine as primary contributors to the earthy and sweet pepper notes in farmhouse Cheddar cheese (Neta et al., 2008). Damascenone ranks among the most prevalent plant-derived aroma compounds, frequently appearing in processed foods and beverages. It is particularly significant in alcoholic beverage production, notably during wine fermentation (Rajini et al., 2011; Sefton et al., 2011). Following fermentation, the aromatic contributions of 2-sec-butyl-3-methoxypyrazine and dammarone in EL bark increased by 2.65% and 0.27%, respectively. This may result from microbial metabolism promoting pyrazine compound synthesis and terpenoid release (Rajini et al., 2011) corresponding to Wang et al.'s observation that inoculating *Lactobacillus* and *Bacillus* strains enhances pyrazine synthesis during fermented liquor production (Wang et al., 2025b). This indicates fermentation intensifies the green leaf and fruity notes in EL. Conversely, the earthy aroma component 2-methylisoborneol (ACI) decreased from 17.93% to 15.87%. Furthermore, the aroma contributions of the fatty-tasting compounds trans-2, cis-6-nonenal, 2,4-decadienal, and 2-nonenal all decreased post-fermentation. Additionally, the earthy-tasting 1-octen-3-one, the sulfurous-tasting dimethyl trisulphide, the fatty-smelling trans-2, cis-6-nonenal and 2,4-decadienal all decreased after mixed-microbe fermentation. This indicates that mixed-microbe fermentation can reduce earthy, pungent, and fatty odors in EL, thereby improving their flavor profile. The 2.06% reduction in the contribution of the musty-smelling 2-methylisoborneol suggests that the fermentation process may have diminished the bioavailability of borneol-type compounds through microbial redox reactions. Previous studies have demonstrated BS exhibits favorable degradation activity toward 2-methylisoborneol (Ma et al., 2015). Consequently, it is hypothesized that *Bacillus subtilis* played a role during the fermentation of EL, degrading a portion of 2-methylisoborneol and thereby reducing the earthy and musty odors in the EL.

**Table 5:** Odor, OAV and ACI values of key characteristic volatile compounds in EL and FEL.

Number	Category	Metabolite	Molecular formula	Odor	Odor threshold	EL (OAV)	EL (ACI)	FEL (OAV)	FEL (ACI)
1	Nitrogen-containing organic compounds	2-sec-Butyl-3-methoxypyrazine	C_9_H_14_N_2_O	Grass; vegetal notes	0.000001	6018516.90	35.08	6711908.64	37.73
2	Ketones	Damascenone	C_13_H_18_O	Apple; rose; honey; sweet	0.00000142–0.00000252	5264729.11	30.89	5543359.94	31.16
3	Terpenes	2-Methylisoborneol	C_11_H_20_O	Earthy; musty	0.0000022–0.0000039	3055415.90	17.93	2824306.20	15.87
4	Ketones	1-Octen-3-one	C_8_H_14_O	Mushroom; metallic; earthy	0.000007	899288.04	5.28	878103.42	4.94
5	Organic sulfur compounds	Dimethyl trisulfide	C_2_H_6_S_3_	Sulfurous; rotten vegetable	0.000008	800412.01	4.70	811914.76	4.56
6	Aldehydes	trans-2, cis-6-Nonadienal	C_9_H_14_O	Cucumber; gherkin; fatty	0.00002	355482.33	2.09	332656.68	1.87
7	Aldehydes	2,4-Decadienal	C_10_H_16_O	Fried; rancid fat	0.00003–0.00005	210454.43	1.24	201695.21	1.13
8	Terpenes	beta-Ionone	C_13_H_20_O	Woody; berry; violet	0.0000579–0.000104	121214.12	0.71	124893.64	0.70
9	Organic sulfur compounds	Dimethyl disulfide	C_2_H_6_S_2_	Onion; garlic; rotten; herbaceous; sulfurous	0.00006	98320.69	0.58	99525.03	0.56
10	Aldehydes	2-Nonenal	C_9_H_16_O	Rich in fat; cardboard	0.000065	95827.53	0.56	93187.68	0.52

Differential metabolite analysis further elucidated the regulatory network governing flavor compounds during fermentation. Among the 317 differentially expressed metabolites identified with VIP > 1 and p < 0.05, olfactory activity analysis was conducted (Figure 7D). Among these, 2-methylisoborneol emerged as the predominant odor source within the differential metabolites. However, following mixed-bacteria fermentation, both its odor activity value and aroma contribution decreased, indicating significant sensory implications for optimizing EL flavor.

### 3.7 Mechanism of mixed-bacteria fermentation

**Figure 8:**
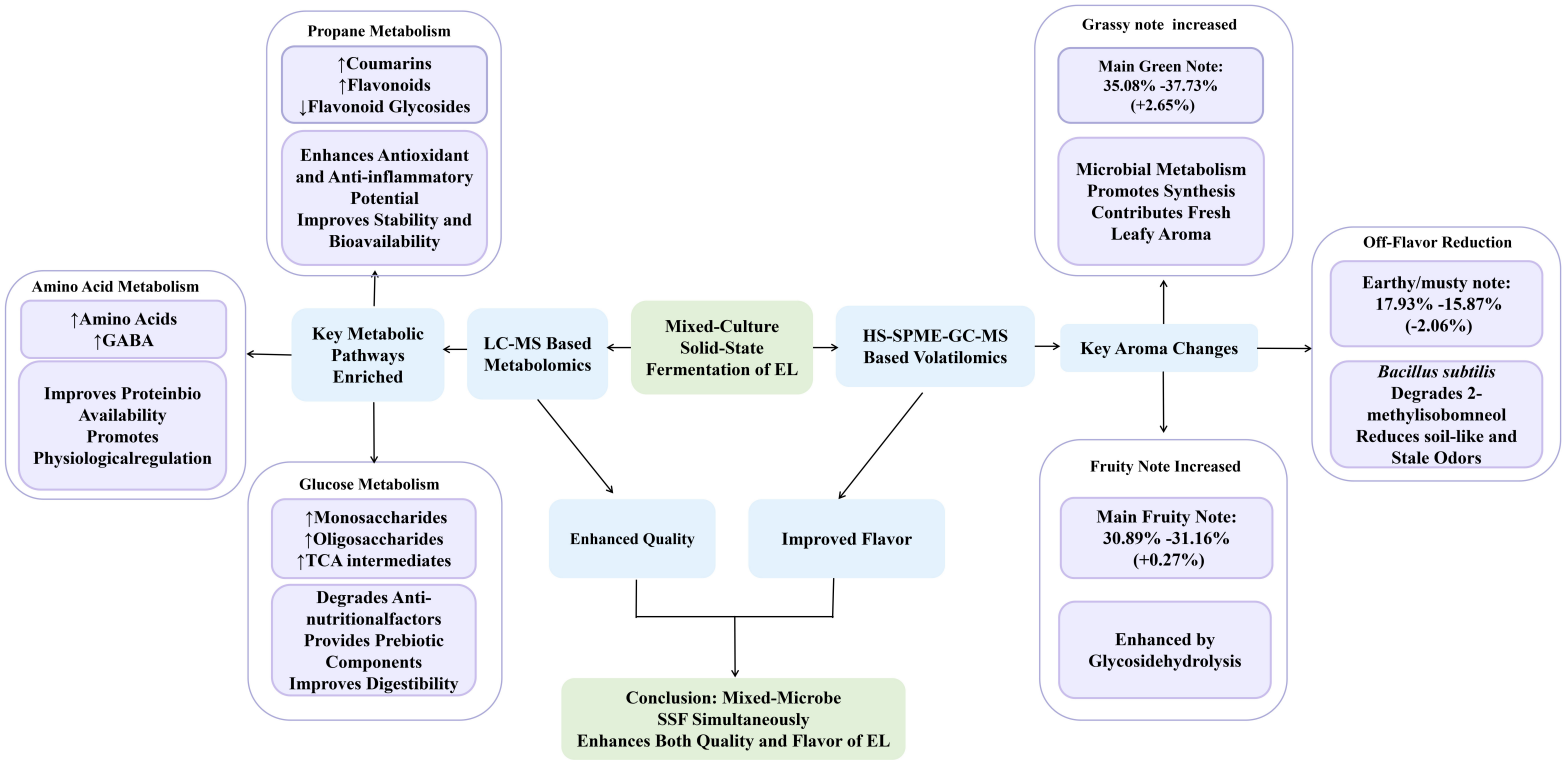
Schematic diagram illustrating the dual enhancement of EL by mixed-culture SSF: LC-MS reveals quality improvement through metabolic pathway activation, while HS-SPME-GC-MS shows flavor optimization through aroma profile modulation.

In this study, mixed-culture SSF of EL was carried out using *Lactiplantibacillus plantarum* and *Bacillus subtilis*, systematically revealing the mechanism by which fermentation drives quality and flavor improvement at the metabolic level. This integrated regulatory network is summarized in Figure 8. Untargeted metabolomics analysis indicated that microbial synergistic metabolism during fermentation significantly remodeled the metabolic network of EL. Through LC-MS untargeted metabolomics analysis, a total of 335 differentially expressed metabolites were identified from 1,698 non-volatile metabolites, primarily including key bioactive components such as amino acids, peptides, flavonoids and alkaloids. Further pathway enrichment analysis revealed specific activation of phenylpropanoid biosynthesis, amino acid metabolism, and carbohydrate metabolism pathways. Among these, the enhanced metabolic flux of phenylalanine promoted the biosynthesis and accumulation of downstream active phenylpropanoids such as GPA and CA, thereby functionally enhancing the quality of EL at the molecular level. Regarding flavor regulation, volatile metabolomics based on HS-SPME-GC-MS identified 317 differential metabolites from 827 volatile compounds. The redirection of metabolic flux led to an increased contribution of characteristic grassy (2-sec-butyl-3-methoxypyrazine) and fruity (damascenone) aroma components, while key off-flavor substances such as the earthy and musty 2-methylisoborneol were significantly degraded via microbial transformation pathways. Electronic tongue analysis further validated this metabolic remodeling effect at the sensory level, showing a decrease in bitterness response and an increase in umami response, indicating that fermentation effectively modulates the metabolic balance of taste-active compounds and comprehensively optimizes the sensory attributesof EL.

## 4 Conclusions

This study examined the bioactive components and metabolomic profile of EL following SSF using a mixed culture of *Lactiplantibacillus plantarum* and *Bacillus subtilis*. The results demonstrate that mixed-culture SSF significantly modulated the bioactive composition of EL. Untargeted metabolomics analysis indicated that differential metabolites were predominantly amino acids, peptides and their derivatives, carbohydrates, flavonoids, alkaloids, organic acids, and vitamins. These alterations collectively underscore the central role of fermentation in nutrient degradation and the transformation of bioactive compounds. Key metabolic pathways involved were phenylpropanoid biosynthesis, amino acid metabolism, starch and sucrose metabolism, as well as the biosynthesis of multiple secondary metabolites. Furthermore, the primary characteristic aromas of EL were identified as 2-sec-butyl-3-methoxypyrazine, dammarene, 2-methylisoborneol, 1-octen-3-one, trisulphide dimethane, trans-2, cis-6-nonadienal, and 2,4-decadienal. Following fermentation, the aroma contributions of 2-sec-butyl-3-methoxypyrazine increased from 35.91% to 38.31%, and that of dammarene from 38.31% to 32.46%, whereas the contribution of 2-methylisoborneol decreased from 17.93% to 11.85%. Differential metabolite analysis identified 317 volatile differential metabolites, among which the earthy and musty-smelling 2-methylisoborneol identified as the key volatile compound responsible for the odor differences in FEL. This study demonstrates that SSF of EL using a mixed bacterial culture effectively enhances bioactive constituents and flavor profiles, while also elucidating the underlying transformation mechanisms. Given that this study was conducted at laboratory scale with a single batch of EL, the reproducibility and scalability of the findings require validation under industrial conditions. Moreover, this work only investigated the combined fermentation of *Lactiplantibacillus plantarum* and *Bacillus subtilis*, whether the conclusions can be extended to other microbial consortia still requires further exploration. Future studies should systematically investigate the functional roles of differential metabolites in determining EL quality and flavor, optimize fermentation parameters for industrial-scale production. Advancing these efforts will facilitate the development of value-added products with distinctive flavor profiles and provide stronger scientific support for the advancement of the *Eucommia* industry.

## Data Availability

The original contributions presented in the study are included in the article/Supplementary material, further inquiries can be directed to the corresponding authors.
