# Correction: Mixed-microbe solid-state fermentation modulates the physicochemical and aroma profiles of *Eucommia ulmoides* leaf

**DOI:** 10.3389/fmicb.2026.1924758

**Published:** 2026-07-16

**Authors:** Bingnan Gu, Wei Zhang, Bing Guo, Xin Wang, Faliang Wu, Yuheng Zhang, Hailong Tian, Xingke Li, Jihong Huang

**Affiliations:** 1 Institute of Microbial Engineering, School of Life Sciences, Henan University, Kaifeng, China; 2 Engineering Research Center for Applied Microbiology of Henan Province, Kaifeng, China; 3 Henan Key Laboratory of Synthetic Biology and Biomanufacturing, Kaifeng, China; 4 Henan Sanweigi Food Limited Liability Company, Sanmenxia, China; 5 Agricultral College, Henan University, Kaifeng, China

**Keywords:** *Eucommia ulmoides* leaf, functional ingredients, metabolomics, mixed-bacteria solid-state fermentation, volatile flavor

In the published article, there was a mistake in the **Funding** statement.

The incorrect **Funding** statement is below:

“The author(s) declared that financial support was received for this work and/or its publication. Financial supports by key Research and Development Project of Henan Province (241111111200).”

The correct statement is below:

“The author(s) declared that financial support was received for this work and/or its publication. This work was supported by Henan Provincial Key R&D Special Project (No. 241111110700).”

The original version of this article has been updated.

